# Diagnostic accuracy of handheld cardiac ultrasound device for assessment of left ventricular structure and function: systematic review and meta-analysis

**DOI:** 10.1136/heartjnl-2021-319561

**Published:** 2021-08-06

**Authors:** Sam Jenkins, Samer Alabed, Andrew Swift, Gabriel Marques, Alisdair Ryding, Chris Sawh, James Wardley, Benoy Nalin Shah, Peter Swoboda, Roxy Senior, Robin Nijveldt, Vassilios S Vassiliou, Pankaj Garg

**Affiliations:** 1 Department of Infection, Immunity and Cardiovascular Disease, The University of Sheffield, Sheffield, UK; 2 Cardiovascular and Metabolic Health, Academic Unit of Radiology, University of Sheffield, Sheffield, UK; 3 Cardiology, Norfolk and Norwich University Hospital NHS Trust, Norwich, UK; 4 Cardiology, Wessex Cardiothoracic Centre, University Hospital Southampton, Southampton, UK; 5 LICAMM, University of Leeds, Leeds, UK; 6 Department of Cardiology, Royal Brompton Hospital, London, UK; 7 Vrije Universiteit Amsterdam, Amsterdam, The Netherlands; 8 Second Floor, Norwich Medical School, University of East Anglia, Norwich, UK

**Keywords:** echocardiography, diagnostic imaging, cardiac imaging techniques, meta-analysis

## Abstract

**Objective:**

Handheld ultrasound devices (HUD) has diagnostic value in the assessment of patients with suspected left ventricular (LV) dysfunction. This meta-analysis evaluates the diagnostic ability of HUD compared with transthoracic echocardiography (TTE) and assesses the importance of operator experience.

**Methods:**

MEDLINE and EMBASE databases were searched in October 2020. Diagnostic studies using HUD and TTE imaging to determine LV dysfunction were included. Pooled sensitivities and specificities, and summary receiver operating characteristic curves were used to determine the diagnostic ability of HUD and evaluate the impact of operator experience on test accuracy.

**Results:**

Thirty-three studies with 6062 participants were included in the meta-analysis. Experienced operators could predict reduced LV ejection fraction (LVEF), wall motion abnormality (WMA), LV dilatation and LV hypertrophy with pooled sensitivities of 88%, 85%, 89% and 85%, respectively, and pooled specificities of 96%, 95%, 98% and 91%, respectively. Non-experienced operators are able to detect cardiac abnormalities with reasonable sensitivity and specificity. There was a significant difference in the diagnostic accuracy between experienced and inexperienced users in LV dilatation, LVEF (moderate/severe) and WMA. The diagnostic OR for LVEF (moderate/severe), LV dilatation and WMA in an experienced hand was 276 (95% CI 58 to 1320), 225 (95% CI 87 to 578) and 90 (95% CI 31 to 265), respectively, compared with 41 (95% CI 18 to 94), 45 (95% CI 16 to 123) and 28 (95% CI 20 to 41), respectively, for inexperienced users.

**Conclusion:**

This meta-analysis is the first to establish HUD as a powerful modality for predicting LV size and function. Experienced operators are able to accurately diagnose cardiac disease using HUD. A cautious, supervised approach should be implemented when imaging is performed by inexperienced users. This study provides a strong rationale for considering HUD as an auxiliary tool to physical examination in secondary care, to aid clinical decision making when considering referral for TTE.

**Trial registration number:**

CRD42020182429.

## Introduction

Echocardiography is the first-line imaging modality for assessing cardiac size and function. Indications for transthoracic echocardiography (TTE) as recommended by the British Society of Echocardiography and other international societies include but not limited to: murmur in the presence of cardiac or respiratory symptoms, valvular stenosis or regurgitation, ischaemic heart disease, any suspicion of heart failure (HF) and arrhythmias.^1–3^ TTE allows determination of left ventricular (LV) dysfunction by assessing LV cavity size, wall thickness, valvular appearances and function as well as for the presence of abnormal blood flow within the heart.^4^

The miniaturisation of ultrasound technology has led to the introduction of handheld cardiac ultrasound devices (HUD).^5^ The portability and accessibility of HUD allow for the augmentation of the bedside physical examination. While physical examination remains the primary method for screening cardiovascular disease, the quality and subsequent referral to echocardiography depend on the examiner’s experience and skill, or lack thereof.^6^ HUD therefore can bridge the gap between the physical examination and the more costly and time-consuming departmental TTE.

Several recent studies have assessed the performance of HUD in diagnosing cardiac disease. The aim of this systematic review and meta-analysis is to assess the diagnostic accuracy of HUD to detect LV abnormalities when compared with TTE in both experienced and non-experienced users.

## Methods

The Preferred Reporting Items for Systematic Reviews and Meta-Analyses guidelines and the Cochrane Handbook of Diagnostic Test Accuracy were followed in the study selection, review process and evidence synthesis.^7 8^

### Patient and public involvement

Patients or the public were not involved in the design, or conduct, or reporting, or dissemination plans of our research.

### Eligibility criteria

Any study comparing the diagnostic performance of HUD and TTE was eligible. The index test was any type or size of a HUD performed by operators of any level of experience. The reference standard was TTE performed by experienced imagers. Studies were included if subjects were aged >18 years, sensitivity, specificity, true positive and negative and false positive and negative findings were reported or if diagnostic data could be extrapolated from the results. Studies with a sample size of <20 participants were excluded.

### Search strategy and study selection

MEDLINE (ProQuest, 1946 to 13 October 2020), EMBASE (Ovid, 1974–2020 week 42) were searched on 13 October 2020. No search filters were applied. The full search strategy is available in the online supplemental document. The references of the included studies were also screened for relevant studies.

10.1136/heartjnl-2021-319561.supp1 Supplementary data

Two authors (SJ and PG) screened the titles and abstracts and reviewed full texts for inclusion criteria. Any disagreements were discussed with a third author (SA). Data extraction and risk of bias analysis was performed by two authors (SJ and PG) and disagreements discussed with a third author (SA). Methods for the quality assessment of individual studies are detailed in online supplemental figure 1.

### Statistical analysis

A bivariate random‐effects model was used to obtain the summary point for the sensitivity and specificity and estimate the corresponding 95% CI and prediction regions for all meta‐analyses including four or more studies. The *metandi* command in Stata V.16 (StataCorp, College Station, Texas, USA) was used to perform the analyses and create summary receiver operating characteristics (SROC). The mada package in R was used to calculate the diagnostic ORs (DORs) for each study and the summary DOR for the pooled results with their respective 95% CIs (R Core Team, R: A Language and Environment for Statistical Computing, Vienna, Austria: R Foundation for Statistical Computing, 2020). The Wilson method was used to calculate the CIs for sensitivities, specificities and false positive rates. The Yates correction was used for testing the equality of sensitivities and specificities. The input for the mada command was the number of true positives (TP), false negatives (FN), true negatives (TN) and false postives (FP) for each study.

The effect of operator experience on the effect size of the diagnostic accuracy was assessed in a subgroup analysis and graphically represented in SROC curves comparing the diagnostic accuracy of experienced and inexperienced operators. A meta-regression was also performed to assess the effect of operator experience.

## Results

### Search results

**Figure 1:**
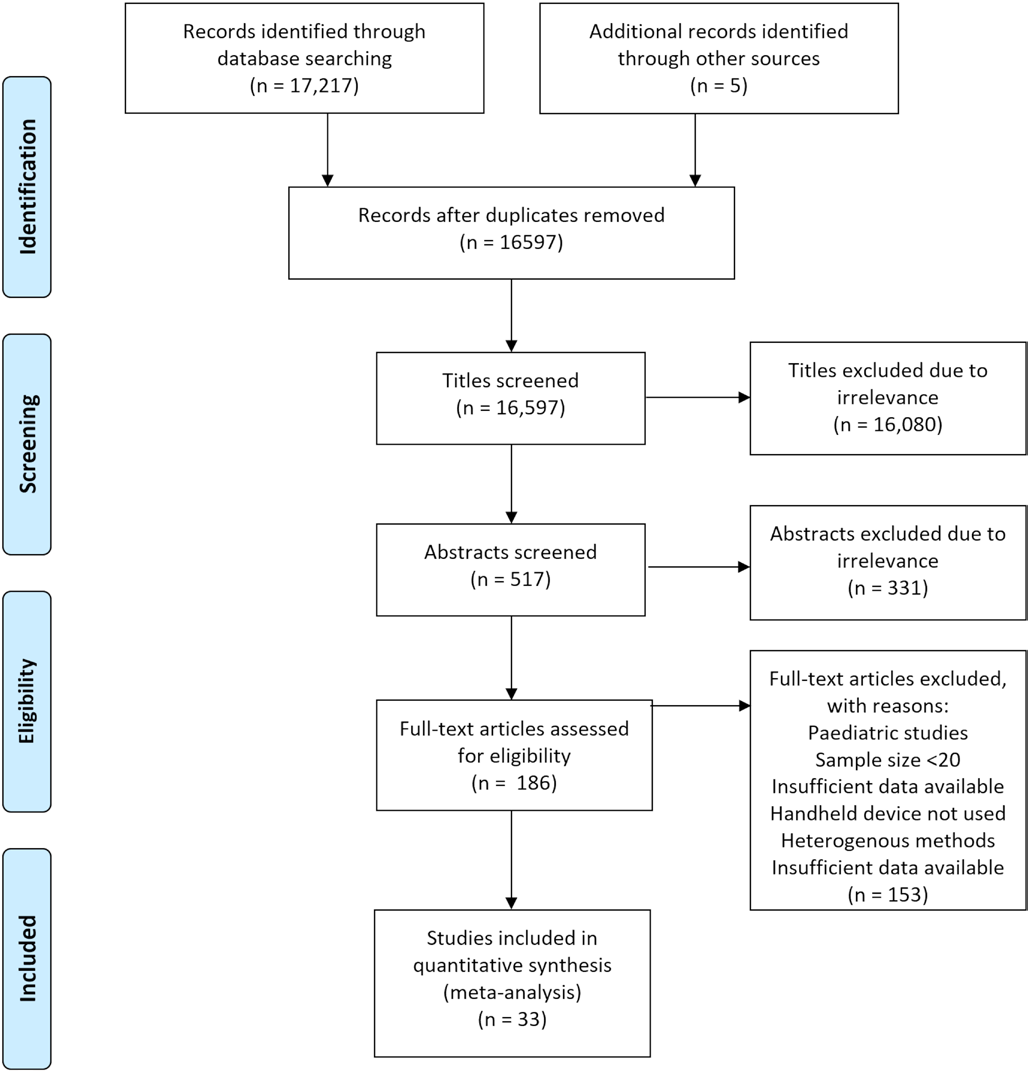
The Preferred Reporting Items for Systematic Reviews and Meta-Analyses flow chart of literature search.

Our comprehensive search identified 33 studies which were incorporated into the meta-analysis. The results of the literature search are outlined in the study flow diagram (figure 1).

### Description of included studies

All studies had a prospective design, with consecutive or random patient selection reported in 21 studies. The studies were published between 2002 and 2019. The majority of studies (22 studies) had a large sample size ≥100 participants, with the largest study by Galasko *et al* 8 recruiting 562 patients.^9^ Individual study data incorporated into the meta-analysis can be found in online supplemental table 1 and a summary graph of the overall risk of bias is shown in online supplemental figure 1.

### Study characteristics

A total of 6062 patients conducted in 13 different countries were included in the meta-analysis. The mean age of patients was 65±5 years with a slight male predominance (54%). Study characteristics are presented in table 1. Study-level HUD data including sensitivity and specificity to predict reduced LV ejection fraction (LVEF), wall motion abnormality (WMA), LV dilatation and hypertrophy (LVH) are reported in the online supplemental materials.

**Table 1:** Characteristics of included studies

Study	Country	Design	Study period	Size	Male (%)	Age, years	Level of experience	HUD
Aldaas *et al* 21	USA	Consecutive	NR	70	50	61±18	Both	Vscan
Alexander *et al* 22	USA	NR	April–November 2000	537	53	59	Inexperienced	Optigo
Andersen *et al* 23	Norway	Random	March–September 2010	108	64	69.1±14	Experienced	Vscan
Biais *et al* 24	France	Consecutive	February–May 2011	151	35	55±20	Experienced	Vscan
Bruce *et al* 9	USA	NR	NR	374	62	66	Both	SonoHeart
Coletta *et al* 25	Italy	Consecutive	April–June 2003	112	57	61±11	Experienced	Optigo
Cullen *et al* 26	USA	Consecutive	2012–2013	190	49	62±17	Experienced	Vscan
DeCara *et al* 27	USA	NR	NR	300	NR	NR	Experienced	Optigo
Fedson *et al* 28	USA	Consecutive	NR	103	NR	NR	Inexperienced	Optigo
Galasko^8^	UK	Consecutive	2000–2001	562	56	62±11	Experienced	Optigo
Ghani *et al* 29	USA	Consecutive	NR	80	51	75±13	Inexperienced	Optigo
Giusca *et al* 30	Romania	Consecutive	NR	56	54	60±12	Inexperienced	Acuson P10
Gulič *et al* 31	Slovenia	Consecutive	NR	200	43	70	Both	Vscan
Khan^32^	USA	Consecutive	2012–2013	240	53	71±17	Experienced	Vscan
Kirkpatrick^33^	USA	NR	NR	63	46	65±16	Inexperienced	Optigo
Kobal *et al* 34	USA	Consecutive	NR	61	62	70±19	Inexperienced	Optigo
Liebo^35^	USA	Consecutive	February–March 2010	97	45	68±17	Both	Vscan
López-Palmero *et al* 36	Spain	NR	July–December 2013	223	42	76	Inexperienced	Vscan
Lucas *et al* 37	USA	Consecutive	March–May 2007	322	53	56±13	Inexperienced	Micromaxx
Lucas *et al* 38	USA	Consecutive	2008–2009	210	55	55	Inexperienced	NR
Martin *et al* 39	USA	Consecutive	2004–2005	354	47	63±19	Inexperienced	Sonosite Elite
Mehta *et al* 40	USA	NR	NR	250	66	61±15	Experienced	Vscan
Mjølstad *et al* 41	Norway	Consecutive	April–June 2011	199	54	66±18	Inexperienced	Vscan
Nilsson *et al* 42	Sweden	NR	2016–2017	100	55	70±12	Inexperienced	Vscan
Olesen^43^	Denmark	NR	NR	260	49	80	Experienced	Vscan
Perez-Avraham *et al* 44	Israel	Consecutive	July–December 2004	85	37	59±14	Inexperienced	Optigo
Razi *et al* 45	USA	Consecutive	NR	50	58	57±17	Inexperienced	Vscan
Ruddox *et al* 46	Norway	NR	2011–2012	303	61	73	Inexperienced	Vscan
Stokke *et al* 13	Norway	Random	February–May 2012	72	72	65±16	Both	Vscan
Vignon *et al* 47	France	Consecutive	NR	55	69	61±16	Experienced	SonoHeart
Vourvouri *et al* 48	The Netherlands	Consecutive	NR	88	64	59±12	Experienced	SonoHeart or Optigo
Wejner-Mik *et al* 49	Poland	Consecutive	NR	87	67	61±16	Experienced	Lumify
Xie *et al* 50	USA	Consecutive	NR	100	55	59±17	Experienced	SonoHeart

HUD, handheld ultrasound devices; NR, not reported.

### Assessment of methodological quality

The majority of studies (19/25) reported a prospective and consecutive or random design. Only one study reported that HUD assessors were unblinded to TTE results while three studies were judged to incorporate a high risk of bias with TTE assessors unblinded to HUD results. There was some concern for bias when time between HUD and TTE was >48 hours. One study was judged to have a high risk of bias as time between HUD and TTE was >7 days.^10^ The detailed results of the quality assessment are outlined in online supplemental figure 2.

### Meta-analysis of HUD indices

Each characteristic was separated into subgroups based on the investigator’s level of experience. Inexperienced operators were those that learnt HUD as part of the study with limited or no prior echocardiography experience. This subgroup included nurses, medical students, residents, general practitioners and inexperienced cardiology trainees. Experienced operators were those who had undergone level II/III echocardiography training or who were stated to be experienced, expert, or trained in echocardiography.

**Figure 2:**
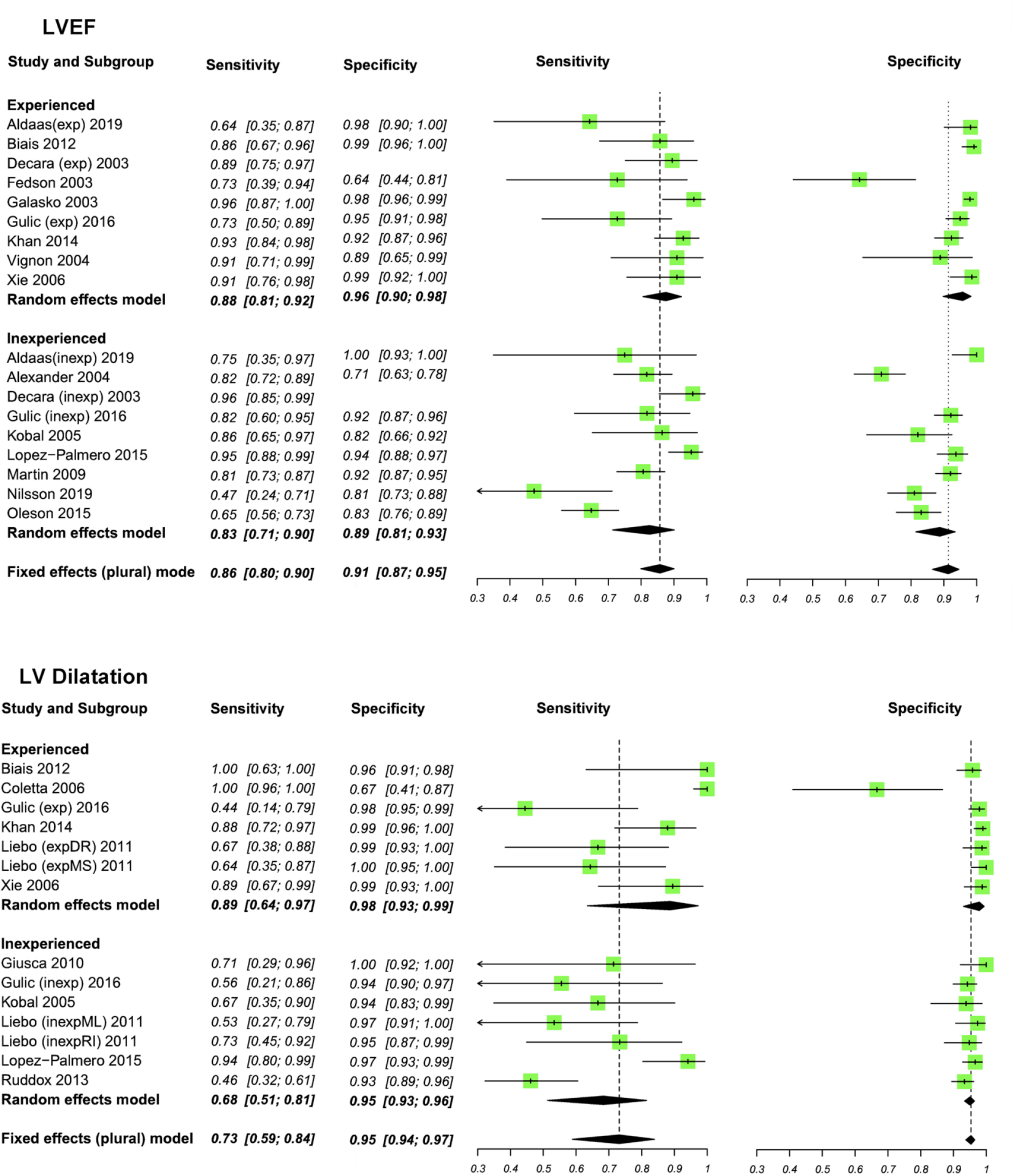
Meta-analyses of left ventricular ejection fraction (LVEF) and left ventricular (LV) dilatation. Sensitivity and specificity (95% CI) values are reported.

**Figure 3:**
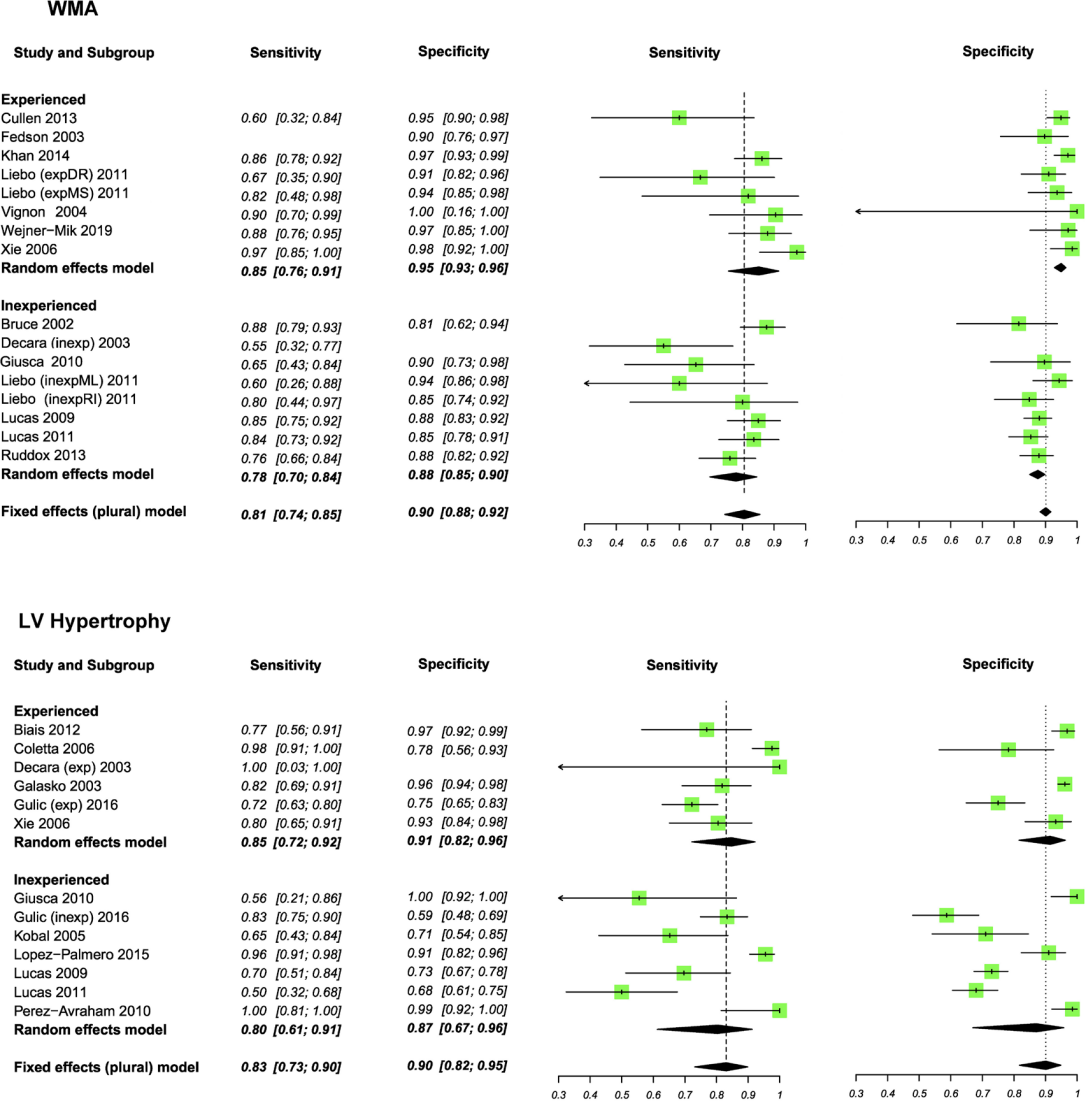
Meta-analyses of wall motion abnormality (WMA) and left ventricular hypertrophy (LVH). Sensitivity and specificity (95% CI) values are reported.

**Figure 4:**
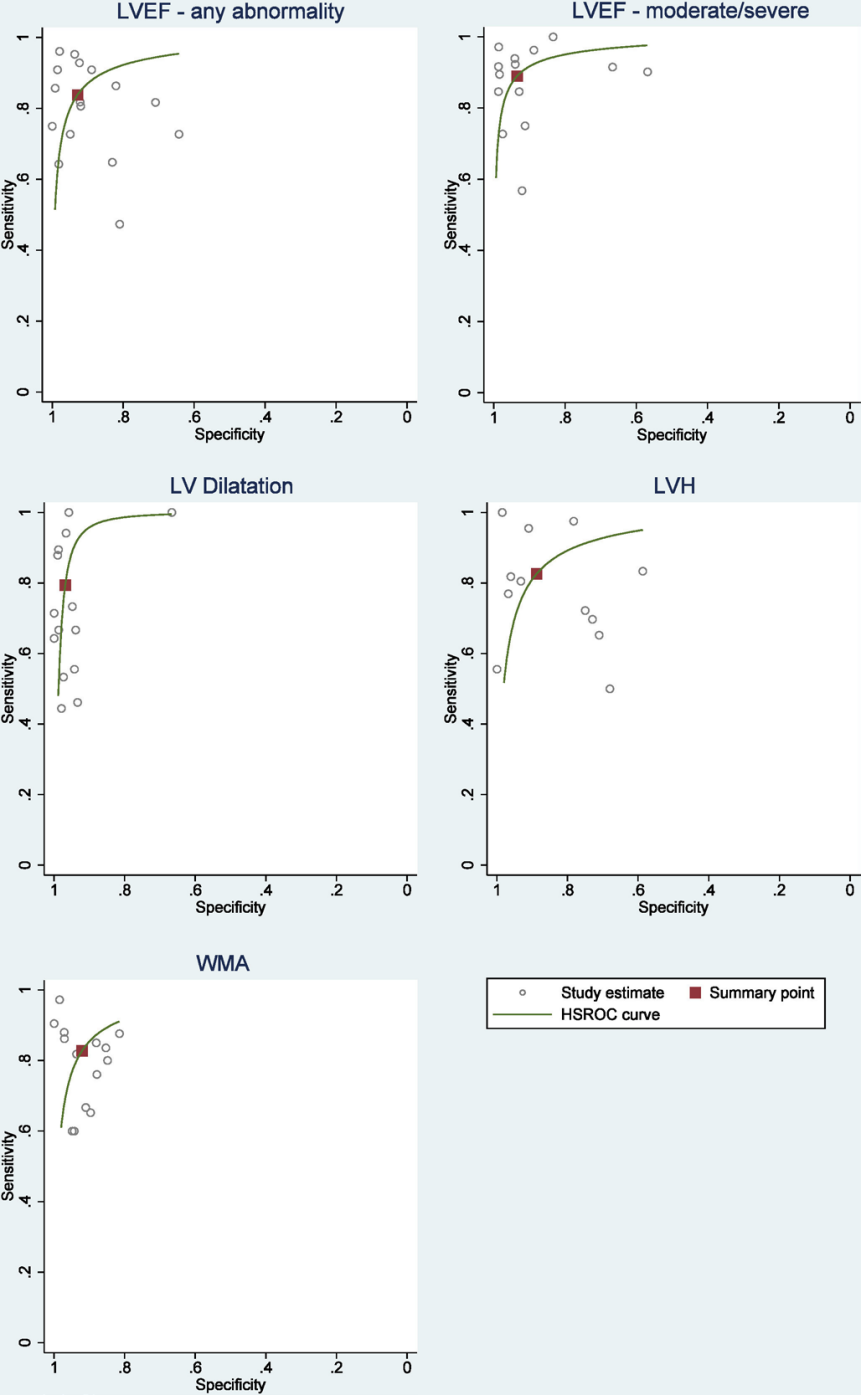
Summary receiver operating characteristic curve (SROC) for the pooled sensitivity and specificity with the summary point for the different handheld ultrasound devices assessments. HSROC, hierarchical summary receiver operator curves; LVEF, left ventricular ejection fraction; LVH, left ventricular hypertrophy; LV, left ventricular; WMA, wall motion abnormality.

**Figure 5:**
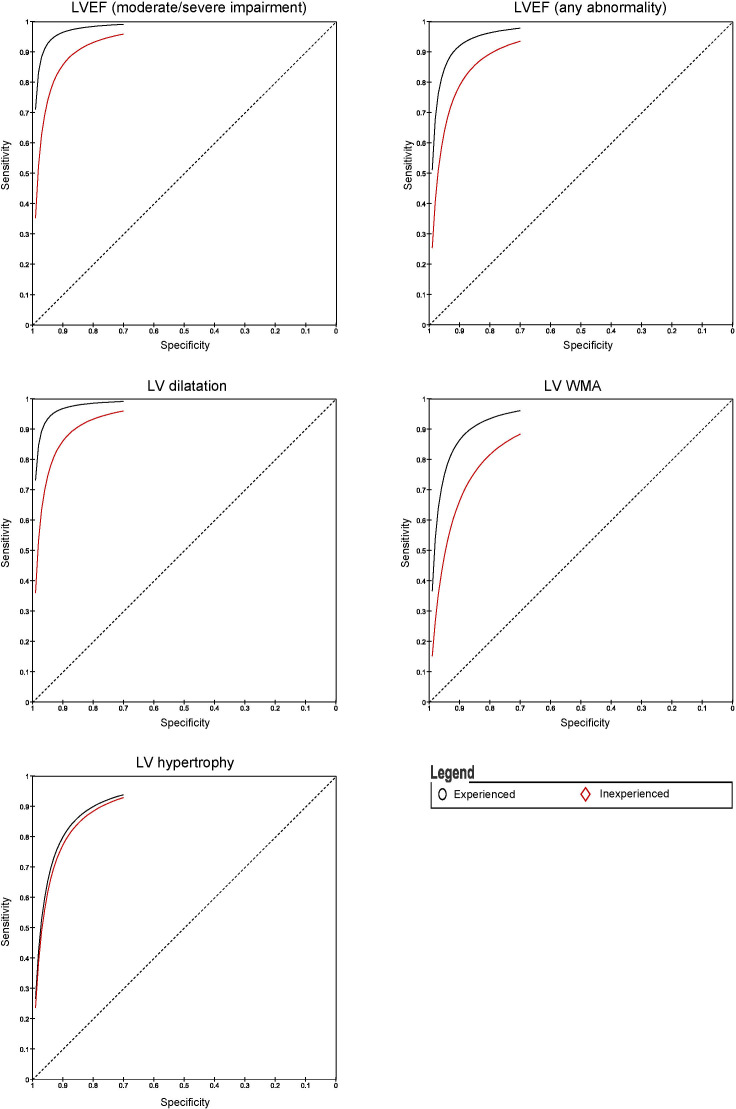
Summary receiver operating characteristic curves for LV parameters comparing effect user experience on handheld ultrasound devices diagnostic accuracy. LVEF, left ventricular ejection fraction; LV, left ventricular; WMA, wall motion abnormality.

Experienced operators could determine reduced LVEF, LV dilatation, WMA and LVH with pooled sensitivities of 88%, 89%, 85% and 85%, respectively (figures 2–4). In inexperienced hands, pooled sensitivities measured were 83%, 68%, 78% and 80%, respectively. Pooled specificities of HUD by experienced users compared with inexperienced users for reduced LVEF, LV dilatation, WMA and LVH measured 96% vs 89%, 98% vs 95%, 95% vs 88% and 91% vs 87%. Online supplemental figure 3 displays a box plot assessing the pooled accuracy of HUD to diagnose moderate/severe LVEF (<45%). SROC curves show an improvement in diagnostic accuracy of reduced LVEF, WMA and LV dilatation when performed by experienced users (figure 5). A summary of the meta-analysis data is provided in table 2.

**Table 2:** Results of meta-analyses comparing the diagnostic accuracy of HUD with first-line TTE

HUD compared with TTE									
Sensitivity %	Total			Experienced			Inexperienced		
	Studies (n)	Prevalence	Sensitivity (95% CI)	Studies (n)	Prevalence	Sensitivity (95% CI)	Studies (n)	Prevalence	Sensitivity (95% CI)
LVEF (any abnormality)	15 (2936)	0.23	86 (80 to 90)	9 (1406)	0.18	88 (81 to 92)	9 (1530)	0.28	83 (71 to 90)
LVEF (moderate/severe)	10 (1611)	0.27	91 (86 to 94)	5 (722)	0.27	93 (89 to 96)	7 (889)	0.27	84 (72 to 92)
WMA	13 (1931)	0.27	81 (74 to 85)	6 (794)	0.26	85 (76 to 91)	7 (1137)	0.28	78 (70 to 84)
LV dilatation	10 (1966)	0.13	73 (59 to 84)	6 (966)	0.17	89 (64 to 97)	6 (1000)	0.09	68 (51 to 81)
LVH	12 (2229)	0.24	83 (73 to 90)	6 (1096)	0.23	85 (72 to 92)	7 (1133)	0.26	80 (61 to 91)
LVEF (any abnormality)	14 (2851)	0.21	91 (87 to 95)	8 (1368)	0.16	96 (90 to 98)	8 (1483)	0.26	89 (81 to 93)
LVEF (moderate/severe)	10 (1611)	0.27	92 (87 to 96)	5 (722)	0.27	96 (87 to 99)	7 (889)	0.27	91 (83 to 95)
WMA	12 (1876)	0.28	90 (88 to 92)	6 (759)	0.27	95 (93 to 96)	6 (1117)	0.28	88 (85 to 90)
LV dilatation	10 (1966)	0.13	95 (94 to 97)	6 (966)	0.17	98 (93 to 99)	6 (1000)	0.09	95 (93 to 96)
LVH	11 (2228)	0.25	90 (82 to 95)	5 (1095)	0.23	91 (82 to 96)	7 (1133)	0.26	87 (67 to 96)

HUD, handheld ultrasound devices; LV, left ventricular; LVEF, LV ejection fraction; LVH, LV dilatation and hypertrophy; TTE, transthoracic echocardiography; WMA, wall motion abnormalities.

The highest diagnostic ORs were in assessing LV dilatation (DOR 96, 95% CI 40 to 229). This result indicates that the odds of a positive result in a patient with LV dilatation is approximately 96 times higher than the odds for a positive result in a person with no LV dilatation. There was a significant difference in the diagnostic accuracy between experienced and inexperienced users in LV dilatation, LVEF (moderate/severe) and WMA. The DOR for LVEF (moderate/severe), LV dilatation and WMA in an experienced hand was 276, 225 and 90, respectively, compared with 41, 45 and 28, respectively, for inexperienced users. The total effect sizes and subgroup differences test comparing the HUD parameters in experienced and inexperienced users are shown in online supplemental table 2. A sensitivity analysis excluding studies that visually assessed LVH and LV dilatation showed that quantitative analysis improves the diagnostic accuracy of HUD. Meta-regression analysis confirmed experience to be a significant factor in the detection of any degree of LVEF dysfunction (p=0.04) and WMA (p=0.01) (online supplemental table 3).

### Heterogeneity

Heterogeneity can be visualised in all the forest plots depicting sensitivity and in the LVEF and LVH plot depicting specificity. Despite there being some overlap of CIs, sampling variation does not completely explain the differences between studies. This apparent heterogeneity can also be observed in the SROC curves for LVEF, WMA and LV dilatation. The increased heterogeneity in the sensitivity plots may be partly explained by fewer patients with the target condition than without. This provides less certainty and results in wider CIs.

A negative correlation between sensitivity and specificity was shown in the meta-analysis of LVEF<45%, WMA and LV dilatation indicating no significant heterogeneity. The correlation coefficients for LVEF (any abnormality) and LVH was positive indicating the presence of possible heterogeneity in the results. We planned to assess the effect of operator experience, pre-existing comorbidities and baseline LV function on the results in a meta-regression covariate analysis. However, only operator experience was sufficiently reported and showed that experience was a significant factor in detecting LVEF, WMA and LV dilatation.

Variation in thresholds likely accounts for some heterogeneity when measuring LVEF (any abnormality), LV dilatation and LVH. Studies with both qualitative and quantitative methodologies were incorporated into the meta-analysis. Quantitative thresholds of LV dilatation ranged from >53 mm to >59 mm, with some studies classifying LV dilatation subject to gender. Similarly, studies measuring LVH were both qualitative and quantitative, with thresholds ranging from >10 mm to >12 mm. A sensitivity analysis excluding qualitative assessment of LVH and LV dilatation showed an improvement in the diagnostic accuracy (online supplemental table 3).

Other sources of heterogeneity may also exist that cannot be assessed including functionality and technological advancement of handheld devices that may allow for more accurate results to be obtained. Some studies reported any abnormality in LVEF as a positive finding without quantifying the results into mild, moderate or severe, which may have led to heterogeneity between studies. However, to limit the effect of the possible heterogeneity we analysed studies reporting LVEF <45% (moderate and severe degrees of LV dysfunction) separately. This analysis allowed for a detailed assessment of HUD in significant disease.

Varying levels of experience using HUD by experienced echocardiographers may account for some heterogeneity. The level and volume of training received by non-experienced users prior to each study was also variable adding to heterogeneity within the non-experienced subgroup.

## Discussion

To the best of our knowledge, this is the first meta-analysis of the diagnostic accuracy of HUD for imaging LV cardiac structural and functional abnormalities and the first to report the impact of user experience. The meta-analysis shows that HUD is both a sensitive and specific method for assessing LV function and morphology when performed by experienced operators. While a basic competence in HUD can be achieved in a relatively short period of time, in clinical practice, a cautious, supervised approach should be applied when inexperienced users are acquiring and interpreting images. This mostly applies to secondary care where the prevalence of disease and the availability of experienced echocardiographers is greatest. Our findings demonstrate that HUD is a valuable bedside tool that can be used to identify those who require further investigation and as a result may lead to a reduction in the number of unnecessary echo referrals (table 3).

**Table 3:** Summary of findings

Review question	Is handheld echocardiography able to accurately diagnose LV dysfunction compared with TTE?			
Population	6062 participants aged 65±5 years with a male predominance of 54% requiring routine referral for TTE			
Setting	Single centres with access to TTE			
Studies	Studies of diagnostic tests			
Quality of evidence	Majority of studies reported consecutive or random sampling, blinding of assessors and short time between HUD and TTE imaging (24–28 hours)			
Pooled results	Sensitivity (95% CI)		Specificity (95% CI)	
	Experienced	Inexperienced	Experienced	Inexperienced
LVEF (any abnormality)	88 (81 to 92)	83 (71 to 90)	96 (90 to 98)	89 (81 to 93)
LVEF (moderate/severe)	93 (89 to 96)	84 (72 to 92)	96 (87 to 99)	91 (83 to 95)
WMA	85 (76 to 91)	78 (70 to 84)	95 (93 to 96)	88 (85 to 90)
LV dilatation	89 (64 to 97)	68 (51 to 81)	98 (93 to 99)	95 (93 to 96)
LVH	85 (72 to 92)	80 (61 to 91)	91 (82 to 96)	87 (67 to 96)

HUD, handheld ultrasound devices; LV, left ventricular; LVEF, LV ejection fraction; LVH, LV dilatation and hypertrophy; TTE, transthoracic echocardiography; WMA, wall motion abnormalities.

Training is required to be able to use HUD competently and therefore the results of our study should be interpreted based on the level of operator experience. The amount of training offered and ability to practise using HUD will also impact the diagnostic outcome that can be expected. Operators with limited training however were less able to detect LV dilatation and WMA, recording pooled sensitivities of 68% and 78%, respectively compared with 89% and 85% achieved by experienced echocardiographers. A positive scan in an experienced hand had a 3–6 times higher odds of showing true LV impairment, LV dilatation or WMA compared with inexperienced operators. Operator experience was not a significant discriminating factor when measuring LVH suggesting that it can be more accurately measured by clinicians with minimal training.

Important logistical points need to be considered including cost-effectiveness, training and accessibility by clinicians. HUD has been shown to be more cost-effective in comparison to TTE and reduces overall costs when compared with physical examination.^6 11^ Despite these potential savings, implementation requires training and frequent revalidation to maintain the clinician’s skill. Didactic and practical sessions are required before clinicians can achieve basic competence in HUD. A minimum of 30 scans has been recommended, however brief training is associated with an increased false-positive rate.^12 13^ This first highlights that HUD performed by inexperienced users should be supervised by experienced echocardiographers as previously mentioned and second, the importance of frequent training and consolidation in echocardiography before allowing users to image patients without supervision. Accessibility of recorded images is therefore a fundamental property of a HUD if sufficient supervision is to be achieved.

Our data suggest that HUD is best positioned at the beginning of the clinical pathway when suspicious of cardiac pathology, thus augmenting the cardiovascular examination. Detecting cardiac disease earlier, when the prevalence of any abnormality is at its lowest, is likely to incur higher rates of false positives. However, HUD should be considered as a method of triaging patients who may require further investigation. HUD should not be considered as a replacement to TTE. HUD can be conducted at the bedside and take <6 min.^14^ It can be argued that this is preferable compared with the potential of unnecessarily having to wait for a more complex scan, which may require the patient to return on a separate day or result in a longer stay in hospital. However, a negative finding on HUD and not proceeding to full TTE risks cardiac abnormalities being missed, a factor which should be considered on an individual patient basis.

Negative results (ie, specificity) can be used with reasonable confidence used to determine normal cardiac physiology. However, positive results need to be interpreted with caution, especially when performed by inexperienced HUD users. Clinical decision making should not be solely guided by the interpretation of HUD images, even by experienced echocardiographers. Instead, these results suggest that HUD should act as an initial diagnostic test to aid decision making on whether further investigation, including TTE, and treatment is required.

Given the excellent sensitivity and specificity particularly seen with more experienced operators, it is likely that the role of HUD will become even more prominent in future clinical practice. We would support earlier teaching and training of HUD and its incorporation into the medical school curriculum, thus providing an important way of ensuring adequate training for all future doctors.

### Strengths and limitations

An extensive literature search was performed. No search filters were used revealing results from an unpublished source, thus minimising the risk of publication bias. To limit the effect of any reporting bias, data were carefully extracted from the results of some studies that did not explicitly state sensitivity and specificity values. The search and data extraction were performed by two authors independently to minimise the bias in the review process.

Half of the included studies are >10 years old and improvement in screen technology, image processing and other advancements might play a pivotal role in improving the diagnostic accuracy of HUD.

The lack of a common threshold for WMA, LV dilatation and LVH means the diagnostic performance may vary between centres. Our results do however show that despite variation in thresholds, the specificity of HUD remains ≥87% for all characteristics. Even if sensitivity is reduced, the use of HUD as an initial diagnostic tool means diagnosing a condition and determining disease severity is not the aim of this test. Any uncertainty when interpreting the image should result in referral for further investigation. Clinical suspicion of HF and measurement of LV filling pressures is an important indication for echocardiography assessment and would be a valuable bedside tool. Clarius is one of the only scanners on the market capable of pulse-wave Doppler (PWD), permitted by the installation of a liquid heating device which prevents overheating.^15^ The current technological capabilities of most HUDs do not include PWD and therefore filling pressures cannot be measured using these devices.^16^ We were therefore unable to analyse sufficient data regarding this variable.

### Future applications

With advancement of technology, LV border tracking and other methods of automatic, device-generated, quantitative measures of ejection fraction may become routinely available.^17^ Incorporation of PWD and continuous-wave Doppler technology into a greater number of available HUDs will also allow for a more extensive range of quantitative cardiovascular assessments to be undertaken at the bedside. Qualitative assessment of valvular heart disease using colour-flow Doppler is available on most HUDs, however is outside the scope of this review. Images may ultimately be sent remotely following acquisition directly to an experienced echocardiographer who can review and interpret images immediately, fast-tracking patients who require further investigation.^18 19^ Further developments in HUD therefore have the potential to revolutionise the bedside cardiovascular examination.

This study highlights the need to further develop methods to bridge the gap between experienced and inexperienced users. Education and introduction of an ultrasound curriculum for medical students and junior doctors can improve understanding of clinical anatomy, develop basic ultrasound skills and later their diagnostic ability.^20 21^ Formulation of a designated HUD training pathway would allow for standardisation of HUD competencies and provide structure to those wishing to advance their experience using HUDs. These suggestions are limited by the financial burden this would incur as well as a shortfall of clinicians adequately trained using HUDs.

## Conclusion

This meta-analysis supports the use of HUD as a powerful modality for predicting LV size and systolic function. HUD diagnostic yield is superior when performed by experienced echocardiographers. Images acquired by an inexperienced operator should be done under direct supervision or validated by a more experienced user. This study provides a strong rationale for considering HUD as an auxiliary tool to the physical examination in secondary care, to aid the clinical decision making when considering referral for TTE.

**Key messages**

**What is already known on this subject?**

- Handheld cardiac ultrasound device (HUD) offers rapid bedside assessment of cardiac morphology and function.
- The diagnostic accuracy of HUD previously reported has shown heterogeneity between studies and its clinical value has yet to be determined.

**What might this study add?**

- To the best of our knowledge, this is the first study to perform a meta-analysis evaluating the diagnostic accuracy of HUD devices to detect cardiac dysfunction and the impact of operator experience on test accuracy.
- This study reports that HUD test accuracy is significantly improved when performed by experienced operators.

**How might this impact on clinical practice?**

- The clinical utility of HUD is rapidly expanding.
- Augmentation of the physical cardiovascular examination using HUD may improve detection of cardiac size and function at the bedside and lead to a reduction in the number of unnecessary departmental transthoracic echocardiography referrals.
- Image interpretation by inexperienced operators should be confirmed by more experienced echocardiographers before clinical decisions and referral for further imaging are made.

## Data Availability

All data relevant to the study are included in the article or uploaded as supplementary information.
