# Supplementary material for: Diagnostic accuracy of handheld cardiac ultrasound device for assessment of left ventricular structure and function: systematic review and meta-analysis
Source: Heart. 2021 Aug 6;107(22):1826–34. doi: 10.1136/heartjnl-2021-319561 (PMC8562308; doi:10.1136/heartjnl-2021-319561)
Supplement: Supplementary data [file heartjnl-2021-319561supp001.pdf]

HUD MA Supplementary Material

Search terms used in MA

Medline

5	(handheld OR hand?held OR pocket?siz* OR hand?carried OR mobile OR bed?side OR bedside).ti,ab	141,288
6	(echocardiogram OR echocardiography OR ultrasound).ti,ab	359,331
7	(5 AND 6)	6,198

EMBASE

1	(handheld OR hand?held OR pocket?siz* OR hand?carried OR mobile OR bed?side OR bedside).ti,ab	183,391
2	(echocardiogram OR echocardiography OR ultrasound).ti,ab	580,544
3	(1 AND 2)	11,019

Table 1. Individual study data incorporated into the meta-analysis.

Study	Parameter	Threshold	Whole Sample Size	Total Number Measured	Total Number Positive	Sensitivity (%)	Specificity (%)	PPV (%)	NPV (%)
**LVEF**									
Aldaas (exp) 2019	LV border detection and tracking	LVEF <50% - At least adequate image quality	70	56	8	75	98	N/A	N/A
Aldaas (inexp) 2019	LV border detection and tracking	LVEF <50% - At least adequate image quality	70	56	8	75	100	100	96
Alexander 2004	Visual estimation of LV systolic function	LVEF <55%	537	533	223	82	71	67	85
Andersen 2011	Qualitative assessment	SE>=moderate pathology	108	108	35	97	99	97	99
Biais 2012	Visual estimation of LV systolic function	LVEF <50%	151	151	28	86	99	96	97
Decara (exp) 2003	Qualitative assessment	LV dysfunction	300	149	38	89	N/A	N/A	N/A
Decara (inexp) 2003	Qualitative assessment	LV dysfunction	300	151	47	97	N/A	N/A	N/A
Fedson 2003	Qualitative assessment	>=mild	103	39	11	73	64	44	86
Galasko 2003	Visual estimation of LV systolic function	LVEF <50%	562	531	51	96	98	83	99.6
Ghani 2006	Visual estimation of LV systolic function	LVEF <40%	80	73	16	75	91	71	93
Gulic (exp) 2016	Qualitative assessment	Any reduction in LVEF	200	200	22	72	94	64	97
Gulic (inexp) 2016	Qualitative assessment	Any reduction in LVEF	200	200	22	84	92	58	98
Khan 2014	Visual estimation of LV systolic function	Normal >=55%, moderate 35%-<55%, severely reduced <35%	240	239	70	93	92	84	97

Kirkpatrick 2005	Visual estimation of LV systolic function	LVEF <40%	63	63	3	100	83	23	100
Kobal 2005	Visual estimation of LV systolic function	LVEF <50%	61	61	22	86	82	73	91
Liebo (expDR) 2011	Visual estimation of LV systolic function	LVEF <45%	97	91	13	85	99	92	97
Liebo (expMS) 2011	Visual estimation of LV systolic function	LVEF <45%	97	90	12	92	99	92	99
Liebo (inexpRI) 2011	Visual estimation of LV systolic function	LVEF <45%	97	97	13	85	93	65	98
Liebo (inexpML) 2011	Visual estimation of LV systolic function	LVEF <45%	97	92	11	73	98	80	96
Lopez-Palmero 2015	Qualitative assessment	Normal or depressed	223	212	85	95.2	93.7	91	96.7
Martin 2009	Qualitative assessment	Normal or abnormal	354	336	124	81	92	85	89
Mehta 2014	Qualitative assessment	LVEF<40%	250	250	54	96	89	70	99
Mjolstad 2013	Systolic excursion of AV plane and visual estimation of LV systolic function	LVEF <45% = moderate+	199	129	30	92	94	80	98
Nilsson 2019	Visual estimation of LV systolic function	LVEF<50%	100	140	19	47	81	28	91
Oleson 2015	Qualitative assessment	LVEF<50%	260	255	125	65	83	79	71
Razi 2011	Visual estimation of LV systolic function	LVEF <40%	50	50	33	94	94	97	88
Ruddox 2013	Visual estimation of LV systolic function	LVEF<40%	303	283	81	57	92	74	84
Stokke (inexp) 2014	Visual estimation of LV systolic function	LVEF <45%	72	105	61	90	57	74	81

Stokke (exp) 2014	Visual estimation of LV systolic function	LVEF <45%	72	101	59	92	67	79	85
Vignon 2004	Not specified	Not specified	55	42	22	91	89	91	89
Vourvouri 2003	Visual estimation of LV systolic function	LVEF <40%	88	82	19	89	98	94	97
Xie 2006	Not Specified	LVEF<50%	100	100	33	91	99	97	96
**WMA**									
Bruce 2002	Qualitative assessment	Present	374	124	97	88	82	94	65
Cullen 2013	Based on 16-segment model	Present	190	190	15	60	95	50	97
Decara (exp) 2003	Qualitative assessment	Present	300	149	14	79	N/A	N/A	N/A
Decara (inexp) 2003	Qualitative assessment	Present	300	151	20	55	N/A	N/A	N/A
Fedson 2003	Qualitative assessment	Present	103	39	0	N/A	90	N/A	N/A
Giusca 2010	Not Specified	Present	56	52	23	65.2	89.5	76.5	83.3
Khan 2014	Lack of normal systolic thickening or translational motion towards centreline	Present	240	232	94	86	97	95	91
Liebo (expDR) 2011	Lack of normal systolic thickening or translational motion towards centreline	Present	97	90	15	67	91	53	95
Liebo (expMS) 2011	Lack of normal systolic thickening or translational motion towards centreline	Present	97	74	11	82	94	69	97
Liebo (inexpRI) 2011	Lack of normal systolic thickening or translational motion towards centreline	Present	97	76	10	80	85	44	97

Liebo (inexpML) 2011	Lack of normal systolic thickening or translational motion towards centreline	Present	97	80	10	60	94	60	94
Lucas 2009	Abnormal wall movement/thickening during systole	Present	322	314	80	85	88	71	95
Lucas 2011	Abnormal wall movement/thickening during systole	Present	210	210	67	84	85	73	92
Ruddox 2013	Visual estimation	Present	303	261	96	76	88	79	86
Vignon 2004	Not specified	Not specified	55	23	21	90	100	100	50
Wejner-Mik 2019	Visual estimation	Present	87	85	50	88	97	98	86
Xie 2006	Not Specified	Present	100	100	36	97	98	97	98
**LV Dilation**									
Biais 2012	Qualitative assessment	Yes/No SE=LV dilation >55mm	151	151	8	94	96	57	100
Coletta 2006	LVEDD	Normal 30mm/m2	112	105	87	100	65	93	100
Giusca 2010	LV diameter	>59mm	56	52	7	71	100	100	95.7
Gulic (exp) 2016	Qualitative assessment	Yes/No	200	200	9	44	97	50	97
Gulic (inexp) 2016	Qualitative assessment	Yes/No	200	200	9	55	94	31	98
Khan 2014	LVEDD	>53mm women, >59mm men	240	225	33	87	98	91	98
Kobal 2005	Qualitative assessment	Yes/No SE>56mm	61	61	12	67	94	73	92
Liebo (expDR) 2011	LVEDD	>53mm women, >59mm men	97	92	15	67	99	91	94
Liebo (expMS) 2011	LVEDD	>53mm women, >59mm men	97	93	14	64	100	100	94

Liebo (inexpRI) 2011	LVEDD	>53mm women, >59mm men	97	92	15	73	95	73	95
Liebo (inexpML) 2011	LVEDD	>53mm women, >59mm men	97	90	15	53	97	80	91
Lopez-Palmero 2015	LVEDD	>53mm women, >59mm men	223	212	34	94	97	84.2	98.8
Ruddox 2013	LVEDD	Not specified	303	293	52	46	93	60	89
Xie 2006	LVEDD	LVEDD >=55mm	100	100	19	89	99	94	98
**LVH**									
Biais 2012	Qualitative assessment	Yes/No SE=IVS>13mm	151	151	26	77	97	83	95
Coletta 2006	IVS	Normal 12mm	112	105	82	97	79	94	88
Coletta 2006	PW	Normal 12mm	112	105	93	97	70	96	74
Decara (exp) 2003	Qualitative assessment	Severe	300	149	1	100	N/A	N/A	N/A
Galasko 2003	IVS or PW	>=13mm	562	540	31	94	93	45	99.6
Galasko 2003	IVS or PW	>=12mm	562	540	55	82	96	69	98
Giusca 2010	End-diastolic thickness of interventricular septum	>=11mm	56	52	9	56	100	100	91.5
Gulic (exp) 2016	Qualitative assessment	Yes/No SE=IVS>14mm	200	200	108	72	75	77	70
Gulic (inexp) 2016	Qualitative assessment	Yes/No SE=IVS>14mm	200	200	108	83	58	70	75
Kobal 2005	Qualitative assessment	Yes/No SE=IVS/PW>=12mm	61	61	23	65	71	58	77
Lopez-Palmero 2015	IVS or PW	>10mm	223	212	134	96	91	94.8	92.2
Lucas 2009	Posterior/septal wall thickness	>=12mm	322	314	33	70	73	23	95
Lucas 2011	Posterior/septal wall thickness	>=1.4 (M), >=1.3 (F)	210	209	34	50	68	23	88

Perez-Avraham 2010	IVS or PW	IVS>=11.7, PW>=9.8	85	85	18	100	99	95	100
Senior 2004	LVMI	>134gm2/m2 (M), 110 (F)	189	179	46	72	91	73	90
Vourvouri 2002	LVMI	>134gm2/m2 (M), 110 (F)	100	100	18	83	96	79	96
Xie 2006	Septal wall thickness	>=12mm	100	100	41	80	93	89	87

We extracted and collated data using a standardised, agreed upon, data extraction form. Data collected included:

1. Methods:
    - Study design
    - Total duration of study
    - Study setting
    - Date of study
    - Country of study
2. Participants:
    - Number included and analysed for the index and reference test
    - Mean age
    - Gender
    - Inclusion criteria
    - Exclusion criteria.
3. Index test:
    - Type of HUD
    - Experience of operators
    - Number of operators
    - Time between index and reference test
4. Reference Standard:
    - Type of standard echocardiography
    - Experience of operators
    - Number of operators
5. Outcomes: LV parameters including function, dilatation, wall motion abnormality and hypertrophy.
6. Thresholds:
    - LVEF
        - Mild = LVEF 45-55%
        - Moderate/severe = LVEF <45%
    - LV dilatation
        - LVEDD >53mm
    - WMA
        - Present/absent
    - LVH
        - Interventricular septum/posterior wall >10mm
7. Diagnostic data:

- Sensitivity/specificity/PPV/NPV
- TP/TN/FP/FN
- Prevalence
- Total number measured

Table 2. Diagnostic odds ratios for HUD cardiac assessment.

Characteristic	DOR Overall (CI)	DOR Experienced (CI)	DOR Inexperienced (CI)	p-value
**LVEF (any abnormality)**	58.63 (26.11-131.63)	131.24 (37.75-456.22)	28.11 (11.19-70.56)	0.051
**LVEF (moderate/severe)**	88.55 (38.88-201.68)	276.02 (57.70-1320.41)	41.45 (18.34-93.72)	0.035
**WMA**	40.81 (25.19-66.10)	89.97 (30.56-264.86)	28.34 (19.76-40.63)	0.047
**LVH**	26.69 (11.27-78.25)	54.92 (13.96-216.08)	18.17 (5.05-65.41)	0.248
**LVH (quantitative only)**	54.69 (11.99-249.45)	96.59 (50.81-183.59)	39.48 (4.81-323.90)	0.436
**LV Dilatation**	95.81 (40.12-228.85)	224.63 (87.38-577.51)	44.84 (16.38-122.77)	0.022
**LV Dilatation (quantitative only)**	142.29 (42.94-471.48)	405.51 (143.18-1148.50)	62.41 (14.30-272.40)	0.042

Table 3. Heterogeneity assessment and evaluation of operator experience on the diagnostic performance of HUD.

HUD Parameter	Heterogeneity Assessment	Operator Experience	
	Correlation coefficient between log Sp and log SN	Meta-regression covariate coefficient	P value
LVEF	0.3	-1.64	0.04
LVEF <40	-0.14	-1.65	0.04
LV dilatation	-0.39	-0.09	0.88
LVH	0.39	-1.08	0.31
WMA	-0.06	-1.055	0.01

Contrary to intervention meta-analyses, the I^2 statistic is not a reliable indicator for heterogeneity in diagnostic test accuracy reviews and therefore was not assessed(1). Instead, heterogeneity can be analysed by estimating the correlation between sensitivity and specificity(2). In a normal ROC curve, an increase in sensitivity is off-set by a decrease in specificity and therefore they have a negative correlation. A correlation coefficient larger than zero indicates possible heterogeneity. The correlation coefficient of the logit-transformed sensitivity and specificity was calculated for all variables.

The sensitivity and specificity are proportion data and limited between the lower and upper limits of 0 and 1. To apply the correlation statistics and meet its distribution assumption, the proportion data are logit-transformed to release the upper and lower limits of the sensitivity and specificity data(2).

We planned to assess the effect of operator experience, pre-existing comorbidities and baseline LV function on the results in a meta-regression covariate analysis. However, only operator experience was sufficiently reported and showed that experience was a significant factor in detecting LVEF, WMA and LV dilatation.

Domain	Risk of bias (%)		
	High	Unclear	Low
**Flow and Timing**	3	27	70
**Reference Standard**	9	15	76
**Index Test**	3	15	82
**Patient Selection**	0	21	79

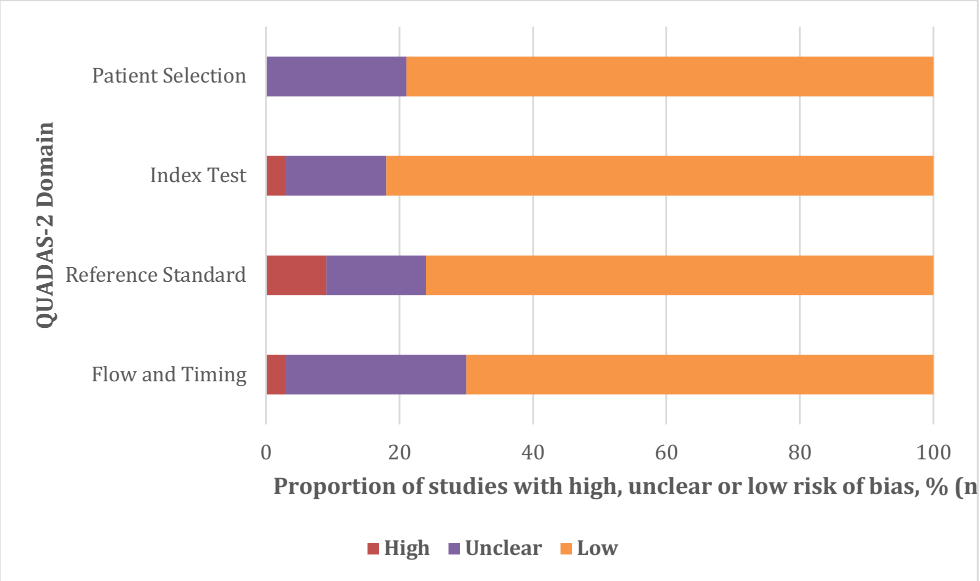

Figure 1. Tabulated and graphical displays for QUADAS-2 results.

The risk of bias was qualitatively assessed based on the domains of the Quality Assessment of Diagnostic Accuracy Studies (QUADAS)-2 tool. Parameters evaluated included random or consecutive recruitment of patients (patient sampling), blinding of assessors to index and reference test, and the time between HUD and TTE (flow and timing). Two reviewers (SJ and PG) independently assessed the risk of bias for each study with disagreements discussed with a third author (SA). Studies categorised as low for all domains were regarded as having a low risk of bias Studies stated as being high or unclear in ≥1 domain were judged to be at risk of bias.

Study	Patient Sampling<br>Random or consecutive recruitment	Index Test<br>HHE Blinded to TTE	Reference Test<br>TTE Blinded to HHE	Flow and Timing<br>Time between HHE and TTE
Aldaas 2019	+	!	!	!
Alexander 2004	!	!	+	+
Biais 2012	+	+	+	+
Bruce 2002	!	+	+	—
Coletta 2006	+	+	+	+
Cullen 2013	+	+	+	+
DeCara 2003	!	+	+	!
Fedson 2003	+	+	!	!
Galasko 2003	+	+	—	!
Giusca 2010	+	+	+	+
Gulic 2016	+	+	!	!
Khan 2014	+	+	+	+
Kobal 2005	+	+	+	+
Liebo 2011	+	+	+	+
Lopez-Palmero 2015	!	+	+	+
Lucas 2009	+	+	+	+
Lucas 2011	+	—	—	+
Martin 2009	+	+	—	+
Nilsson 2019	+	!	!	+
Oleson 2015	+	+	+	+
Perez-Avraham 2010	+	+	+	!
Ruddox 2013	+	+	+	+
Wejner-Mik 2019	+	+	+	+
Xie 2006	+	+	+	+
Vignon 2004	+	+	+	+

Risk of bias

High	—
Some concerns / unclear	!
Low	+

Figure 2. Risk of bias summary for each included study

LVEF <45%
(moderate/severe dysfunction)

Study and Subgroup	Sensitivity	Specificity
**Experienced**		
Andersen 2011	*0.97 [0.85; 1.00]*	*0.99 [0.93; 1.00]*
Liebo (expDR) 2011	*0.85 [0.55; 0.98]*	*0.99 [0.93; 1.00]*
Liebo (expMS) 2011	*0.92 [0.62; 1.00]*	*0.99 [0.93; 1.00]*
Mehta 2014	*0.96 [0.87; 1.00]*	*0.89 [0.84; 0.93]*
Stokke (exp) 2014	*0.92 [0.81; 0.97]*	*0.67 [0.50; 0.80]*
Vourvouri 2003	*0.89 [0.67; 0.99]*	*0.98 [0.91; 1.00]*
**Random effects model**	***0.93 [0.89; 0.96]***	***0.96 [0.87; 0.99]***
**Inexperienced**		
Ghani 2006	*0.75 [0.48; 0.93]*	*0.91 [0.81; 0.97]*
Kirkpatrick 2005	*1.00 [0.29; 1.00]*	*0.83 [0.71; 0.92]*
Liebo (inexpML) 2011	*0.73 [0.39; 0.94]*	*0.98 [0.91; 1.00]*
Liebo (inexpRI) 2011	*0.85 [0.55; 0.98]*	*0.93 [0.85; 0.97]*
Mjolstad 2013	*0.92 [0.75; 0.99]*	*0.94 [0.87; 0.98]*
Razi 2011	*0.94 [0.80; 0.99]*	*0.94 [0.71; 1.00]*
Ruddox 2013	*0.57 [0.45; 0.68]*	*0.92 [0.87; 0.95]*
Stokke (inexp) 2014	*0.90 [0.80; 0.96]*	*0.57 [0.41; 0.72]*
**Random effects model**	***0.84 [0.72; 0.92]***	***0.91 [0.83; 0.95]***
**Fixed effects (plural) model**	***0.91 [0.86; 0.94]***	***0.92 [0.87; 0.96]***

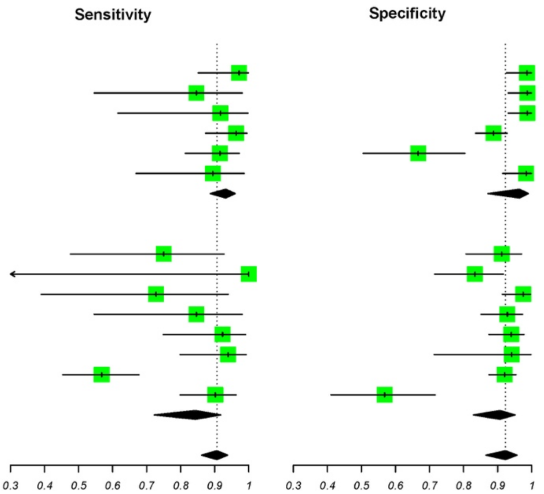

Figure 3. Meta-analyses of LVEF <45%. Sensitivity and specificity [CI] values are reported.

The bivariate method was used to calculated overall sensitivity and specificity and their 95% confidence intervals. The summary estimates of sensitivity and specificity using this method represent the average operating point across studies. The bivariate nature of the input data is maintained throughout the analysis using the bivariate model which allows the calculation of reliable summary estimates. Other advantages of the bivariate method include accounting for the study size, between-study heterogeneity and adjusting for the threshold effect seen when there is a negative correlation between the sensitivity and the specificity of the index test(3).

References

1. Macaskill P, Gatsonis C, Deeks J, Harbord R, Takwoingi Y. Cochrane Handbook for Systematic Reviews of Diagnostic Test Accuracy. :61.

2. Shim SR, Kim S-J, Lee J. Diagnostic test accuracy: application and practice using R software. Epidemiol Health [Internet]. 2019 Mar 28 [cited 2021 Mar 26];41. Available from: https://www.ncbi.nlm.nih.gov/pmc/articles/PMC6545496/

3. Reitsma JB, Glas AS, Rutjes AWS, Scholten RJPM, Bossuyt PM, Zwinderman AH. Bivariate analysis of sensitivity and specificity produces informative summary measures in diagnostic reviews. J Clin Epidemiol. 2005 Oct;58(10):982–90.
